# Chemical Selectivity and Sensitivity of a 16-Channel Electronic Nose for Trace Vapour Detection

**DOI:** 10.3390/s17122845

**Published:** 2017-12-08

**Authors:** Drago Strle, Bogdan Štefane, Mario Trifkovič, Marion Van Miden, Ivan Kvasić, Erik Zupanič, Igor Muševič

**Affiliations:** 1 Faculty of Electrical Engineering, University of Ljubljana, EE dep., Tržaška 25, 1000 Ljubljana, Slovenia; mario.trifkovic@fe.uni-lj.si; 2 Faculty of Chemistry and Chemical Technology, University of Ljubljana, Večna pot 113, 1000 Ljubljana, Slovenia; bogdan.stefane@fkkt.uni-lj.si; 3 J. Stefan Institute, Jamova 39, 1000 Ljubljana, Slovenia; marion.van.midden@ijs.si (M.V.M.); ivan.kvasic@ijs.si (I.K.); erik.zupanic@ijs.si (E.Z.); igor.musevic@ijs.si (I.M.); 4 Faculty of Mathematics and Physics, University of Ljubljana, Jadranska 19, 1000 Ljubljana, Slovenia

**Keywords:** artificial nose, electronic nose, sensor array, vapour trace detection, capacitive microsensors, chemical sensing, explosive detection, gas sensors, signal processing

## Abstract

Good chemical selectivity of sensors for detecting vapour traces of targeted molecules is vital to reliable detection systems for explosives and other harmful materials. We present the design, construction and measurements of the electronic response of a 16 channel electronic nose based on 16 differential microcapacitors, which were surface-functionalized by different silanes. The e-nose detects less than 1 molecule of TNT out of 10^+12^ N_2_ molecules in a carrier gas in 1 s. Differently silanized sensors give different responses to different molecules. Electronic responses are presented for TNT, RDX, DNT, H_2_S, HCN, FeS, NH_3_, propane, methanol, acetone, ethanol, methane, toluene and water. We consider the number density of these molecules and find that silane surfaces show extreme affinity for attracting molecules of TNT, DNT and RDX. The probability to bind these molecules and form a surface-adsorbate is typically 10^+7^ times larger than the probability to bind water molecules, for example. We present a matrix of responses of differently functionalized microcapacitors and we propose that chemical selectivity of multichannel e-nose could be enhanced by using artificial intelligence deep learning methods.

## 1. Introduction

The field of hypersensitive and chemically selective sensors is one of the most important research areas in defense and security technologies as well as in other disciplines, especially considering the development of the Internet of Things (IoT) concept [1]. Solving the problem of chemical selectivity would have a very fundamental influence on further development of sensor array technologies in very different fields. It is thus considered a fundamental research topic of the upmost importance, which is also reflected in the number of publications focused solely on the improvement of the chemical selectivity of detection of individual molecules in the vapour phase.

Sensor systems for trace detection of targeted molecules can be divided into several large families according to their chemical selectivity. Chemically selective sensors based on chromatographic methods are downscaled to microfluidics and micro-structured sensors with appropriate miniaturized columns for gas chromatography [2,3]. These systems show high chemical selectivity, but the digitalization of their measurement readouts is problematic. Arrays of chemically selective MEMS sensors [4] are mechanically rather complicated systems of microcantilevers, which are surface functionalized with receptor molecules. So far, only relatively small systems with a limited number of differential pairs of sensors have been manufactured. These devices are strongly influenced by acoustic and thermal disturbances arising from the environment. Optical sensors based on plasmon nanoparticles [5] use arrays of different plasmon nanoparticles, which can be activated by outside light. Due to large local optical fields amplified by these nanoparticles, the Raman scattering signals of molecules absorbed in these surfaces can be extremely amplified by the SERS effect. Their advantage is high sensitivity and chemical selectivity using specific Raman signals, and their disadvantage is complicated detection. Fluorescent and luminescent sensors use the control of radiated fluorescent light of molecules and nanoparticles and its response to the surface binding of target molecules, such as explosives [6,7]. The advantage of such sensors is their high sensitivity, but the problems are poor chemical selectivity and the optical readout.

We have recently demonstrated a miniaturized vapour trace detector for TNT, based on capacitive detection of adsorbed molecules on the surfaces of chemically functionalized micro-capacitors. The system is implemented in an extremely low-noise measurement detection chip, which can monitor atto-farad changes of the capacitance of chemically modified differential pairs of capacitors with comb-like electrodes. We showed that the system could detect vapour traces of TNT at the concentration of 1 TNT molecule in 10^12^ molecules of carrier gas N_2_ [8]. We also demonstrated that the sensitivity of this method is two orders of magnitude higher compared to the sensitivity of MEMS [9]. In addition to superior sensitivity, the micro-capacitive vapour trace detection method is immune to mechanical noise and almost immune to temperature changes, which makes it an ideal platform for integration with existing CMOS technology.

The overview of the existing studies on chemical selectivity of systems for detection of low target molecules concentration in the atmosphere clearly shows that the main problem is the poor chemical selectivity of all known systems. The chemical selectivity can be improved by increasing the number of sensors with different chemically sensitive surfaces, organizing them into an array scheme. Various examples of sensor arrays were reported in the literature, such as arrays of chemo-resistive polymer-conductor composites [10] and micro-arrays of thin film chemo-resistors with SiO_2_ membrane coatings [11]. On the other hand, we know from biology that chemically selective recognition of targeted vapour molecules in the atmosphere is always based on extremely large number of different sensor cells. For example, mammals have developed up to 1000 odorant receptors [12], which are incorporated on the cilia membrane surface of sensory neurons in the olfactory epithelium. In mice, this olfactory epithelium contains more than 2 million sensory neurons. This leads to a clear conclusion that chemical selectivity of any array system could be improved only by using a very large number of chemically different sensors.

To this end, several studies of different chemically functionalized surfaces to detect the presence of vapours of various explosives were conducted during the last few years. Organosilane-modified silicon surfaces with native SiO_2_ have turned out to be suitable for molecule recognition in many cases [13,14]. The advantages of using modified surfaces for surface sensitive detection of gas analytes are their quick response time, relatively simple and repeatable synthesis of surfaces and the option of post-manufacturing modifications [15]. Modification of various hard surfaces with organic molecules is an important field of material science, which also includes modification of silicon surfaces with a layer of natural oxide SiO_2_. In general, trialcoxy- and trihaloogranosilanes are used for such modifications. Less research has focused to modifications with various acid chlorides, anhydrides, and carboxylic acid esters. Modified (silanised) SiO_2_ surfaces with organosilanes are mostly used in bio-applications, electronics, sensors, photovoltaic systems, etc. [16,17,18]. We have so far successfully investigated different synthetic approaches to modification of silicon surfaces with trialcoxysilaacylamines [19]. Modified surfaces were characterized with different analytical techniques and microscopy [20,21,22].

In this article, we present the design and construction of a 16-channel electronic nose based on differently functionalized micro-capacitor surfaces. Each detection channel is based on a differential capacitor sensor, which consists of two equal micro-capacitors with interdigitated electrodes. They were both covered with a monolayer or a thin layer of a given silane, and this layer was removed on one of the capacitors of the pair. In this way, different surface adsorption of targeted molecules on a differential pair of micro-capacitors is achieved, which causes a slight difference in the capacitance of the two sensors. We present extensive measurements of response of this 16-channels e-nose to different vapours, including different explosives and solvents. We conclude from our measurements that due to complexity of responses, the only feasible strategy for the enhancement of chemical selectivity of multi-channel e-noses is to use artificial intelligence methods and deep learning in the analysis of a large number of parallel signals.

## 2. Materials and Methods

### 2.1. The Sensor

**Figure 1:**
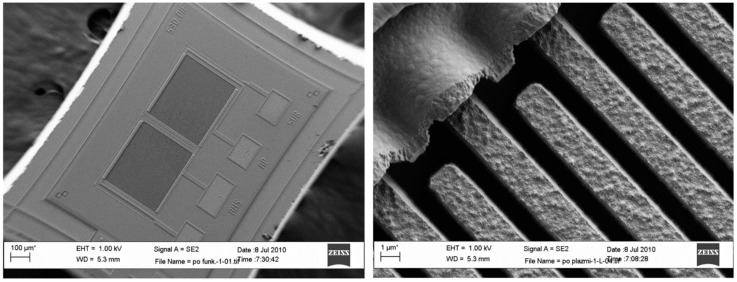
SEM image of a differential pair of COMB micro-capacitor.

The vapour trace detection system is based on planar microcapacitors with inter-digitated electrodes with comb-like structure (COMB capacitors), which are covered with a thin layer of silicon dioxide [8,9]. The finger-like electrodes are made of polysilicon and are 1.5 µm apart and of 2.5 µm height. Each micro-capacitor has outer dimensions of 350 µm × 300 µm and has 51 fingers, each finger having a length of 300 µm. The conductive polysilicon is covered with approximately 10 nm layer of SiO_2_, which provides for good chemical binding of organic molecules, serving as a thin receptor layer for attractive interaction with targeted molecules. Each chip consists of two microcapacitors, shown in Figure 1, which are electrically connected using Al metal lines.

The pair is then surface functionalized with different receptor molecules. After that, the layer of receptor molecules on one of the microcapacitors is removed using Ar^+^ laser treatment, most likely causing thermal decomposition of the organic layer, which loses its ability to preferentially attract targeted molecules to that surface.

After all of the processing, the micro-capacitors do not necessarily have the same capacitance. The capacitance of a single COMB microcapacitor is typically 500 fF and the 3*σ* statistical variation of their matching in a given pair is up to ±10%. This means that difference in capacitance could be as high as 50 fF and should be compensated as much as possible in the detection circuit.

### 2.2. Chemical Functionalization

**Figure 2:**
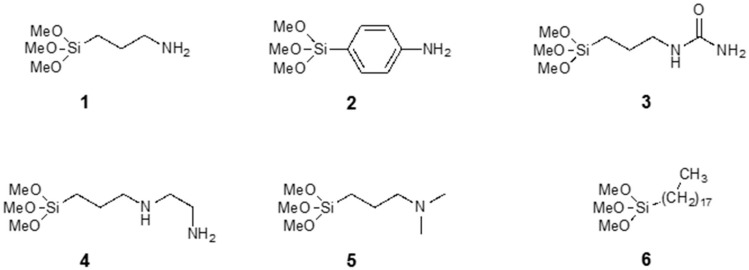
Chemical structures of the silanes used in this work.

**Figure 3:**
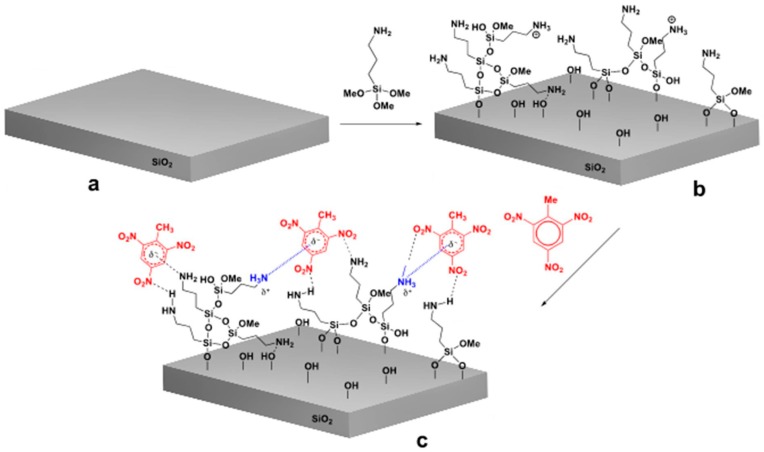
Modification of SiO_2_ surface with APTMS molecules: (**a**) SiO_2_ surface, (**b**) modified SiO_2_ surface (**c**) interaction of analyte with the modified surface.

We modified COMB sensors with six different silanes, shown in Figure 2, where different silanes are marked with numbers 1 to 6. We analysed the surface structure of the organic part of silane molecule and its influence on the detection sensitivity of specific gases. The (3-aminopropyl) trimethoxysilane (APTMS) used in our study is one of the most commonly used aminosilanes in many applications [17,23,24,25,26]. The aminopropyl group in APTMS is flexible and allows a number of intramolecular interactions (see Figure 3). Due to the polymerization of the APTMS molecule and formation of protonated amine function (NH_3_^+^) during modification process [21] we modified sensors also with more rigid aminoarylsilane APhS (*p*-amino-phenyltrimethoxysilane). A thin, ordered and uniform layer was expected in case of APhS modification. Further sensor surfaces were modified with three different aminoalkylsilane derivatives: UPS (1-[3-(trimethoxysilyl)propyl] urea), EDA (*N*-(2-aminoethyl)-3-aminopropyltri-methoxysilane) and DMS ((*N*,*N*-dimethylaminopropyl) trimethoxysilane). UPS is aminopropylsilane functionalized with an amide group; EDA contains an additional aminoethyl group; and the amino group in DMS molecule is functionalized with two methyl groups. Due to their different functional groups, these silanes all have different electronic structures, which could affect the response of the sensor. For comparison, sensors were also modified with the alkylsilane molecule octadecyltrimethoxysilane (ODS). Successful silane treatments with all six precursors were confirmed by X-ray photoelectron spectroscopy (XPS). 

#### 2.2.1. Surface Modifications of the Sensors

The modification of the sensors was carried out in Teflon containers using a 3 mM solution of silanes in anhydrous HPLC grade acetonitrile as solvent (Sigma-Aldrich, St. Louis, MA 01821, USA), dried using CaH_2_ (24 h) and filtered before use over a 0.2 µm filter). 

APTMS (shown in Figure 3), UPS, EDA, DMS and ODS were obtained from Sigma-Aldrich and used as received. APhS was obtained from ABCR Co. (St. Louis, MA 01821, USA) and also used as received. The silanization reaction took place in closed Teflon containers for 6 h at 25 °C. The Teflon containers was purged with nitrogen prior to and during salinization. After completion of the salinization step, the wafers were rinsed with acetonitrile (2×), a mixture of acetonitrile and ethanol = 1:1 (2×), ethanol (2×) and dried at 80 °C for 15 min in a clean oven, to remove the physisorbed silane molecules and any traces of solvent from the surface. The XPS characterization was carried out immediately upon cooling.

#### 2.2.2. Surface Characterization

The chemical composition of the surfaces was determined by X-ray photoelectron spectroscopy (XPS). This analysis was performed with a TFA XPS spectrometer, produced by Physical Electronics Inc. (Chanhassen, MN, USA), equipped with a monochromated Al K_α_ X-ray source (1486.6 eV), under ultra-high vacuum (10^−7^ Pa). The analysed area was 0.4 mm in diameter and the analysed depth was 3–5 nm. During the analysis, survey and high-resolution spectra were recorded. The high-energy resolution spectra were acquired with energy resolution of about 0.6 eV and pass energy of 29 eV. The XPS spectra were processed with the software MultiPak, Ver. 9.5.0. Prior to the spectra processing, the spectra were shifted to the C‒C/C‒H peak in the C 1s core level at a binding energy of 284.8 eV. The accuracy of binding energies was about ±0.2 eV. Quantification of surface composition was performed from XPS peak intensities taking into account the relative sensitivity factors provided by the instrument manufacturer [21,22,23,24,25,26,27]. Two different places were analyzed on each sample and the data were averaged.

#### 2.2.3. Results of Surface Characterization

The surface composition and chemical bonding of the sensors were studied using the XPS technique. The acquired XPS spectra of the uncoated sensor show peaks of carbon, oxygen and silicon. The carbon signal on the surface is probably related to the carbon from the contamination layer. The XPS spectra of the APTMS, APhS, UPS, EDA and DMS modified samples contain, in addition to carbon, oxygen and silicon peaks, also a nitrogen peak, which is related to the structure of the silane molecules. The C, O, Si and N concentrations calculated from the intensities of the XPS peaks are shown in Table 1. From the nitrogen concentration we can estimate the amount of deposited molecules. During the estimation for UPS and EDA, we must take into account half value of nitrogen concentration, since they contain two nitrogen atoms in the structure. The highest number of silane molecules were obtained by using APTMS as precursor. In case of APhS we detected less molecules on the surface and the least intensive interaction between the silane molecules and sensor surface was observed in case of UPS, EDA and DMS. 

**Figure 4:**
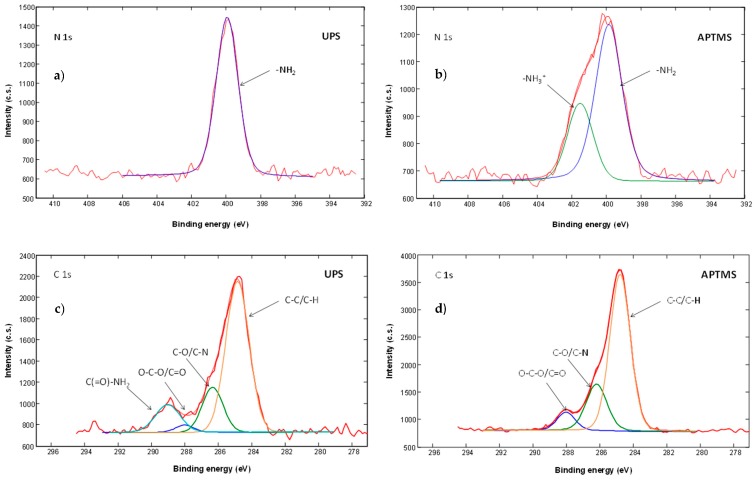
(**a**) High-energy resolution N1s XPS spectra from UPS, (**b**) from APTMS modified sensor surface, (**c**) High-energy resolution C 1s XPS spectra from UPS and (**d**) from APTMS modified sensor surface.

We also studied high-energy-resolution XPS spectra of the modified surfaces. C 1s, O 1s, N 1s and Si 2p high-energy-resolution XPS spectra were acquired. In the obtained spectra we recognized different components related to different types of atomic bonds. For example, C 1s XPS spectra from UPS and APTMS modified surfaces are presented on Figure 4. In the C 1s spectra from UPS sample (Figure 4a) we detected, in addition to C‒C/C‒H at a binding energy of 284.8 eV, C‒O/C‒N at 286.2 eV and O‒C‒O/C=O at 288.1 eV, also peak at a binding energy of 289.0 eV, which corresponds to the amide carbon atom (‒(C=O)‒NH_2_) and confirming successful UPS modification.

We can also recognize the presence of two different components in the N 1s spectra. The component at a binding energy of 399.2 eV corresponds to ‒NH_2_ bonds and the component at a binding energy of 401.0 eV corresponds to –NH_3_^+^ type of bonds. –NH_3_^+^ signal indicates intermolecular and intramolecular interactions of NH_2_ silane group with other molecules (H-bonds, electrostatic interactions and polymerization) or sample surface. N 1s spectra from UPS (Figure 4a) and APhS modified samples are similar and show the presence of only the ‒NH_2_ component. From that we can conclude that most of the APhS and UPS molecules are well oriented on the sample surface, bonded through Si‒O‒Si bonds with the free amino group oriented away from the surface. In case of APTMS (Figure 4b), EDA and DMS we observed in addition to ‒NH_2_ also a –NH_3_^+^ signal. The presence of this –NH_3_^+^ signal indicates an interaction of the molecular amino group with other molecules and/or the surface showing a less ordered silane layer. 

APTMS modified sensor surface is highly sensitive to structurally related nitro-aromatic chemical derivatives such as TNT, 2,6-dinitrotoluene, and nitrotoluene. Based on the results obtained, the interaction model between analyte and APTMS (**1**) modified sensor surface is presented in Figure 4b. As reported by Patolsky et al. [17] the free amino groups, present on the surface of modified sensor, form a reversible charge transfer complex. For example, the negative charge formed on the complexed TNT molecules can be additionally stabilized by neighboring amino groups of aminosilane molecules, consequently leading to more stable complex resulting in enhanced sensitivity of sensor for the nitro-aromatic analytes.

### 2.3. Electronic Detection System of the 16-Channel E-Nose

In this section, we present a multi-channel electronic detection system based on precision electronic measurements in real time of sensor pair capacitance changes. The advantage of this method is its robustness and independence of the environmental influences like pressure, vibration, and temperature [9]. In addition, the measurements are fast, the system is small, cheap if produced in large quantities, it is easy to handle, and it has small power consumption. To be able to detect different target molecules and to improve the selectivity of the complete detection system, we built an array of differently modified sensors together with an integrated, low noise electronic detection system. Each is capable of detecting the change of a capacitance difference in one pair that is smaller than 1 aF/$\sqrt{\mathrm{Hz}}$. With such a detection system, it is possible to detect approximately 1 ppt of TNT target molecules in N_2_ carrier gas.

#### 2.3.1. Sixteen-Channels Vapour Trace Detection System

**Figure 5:**
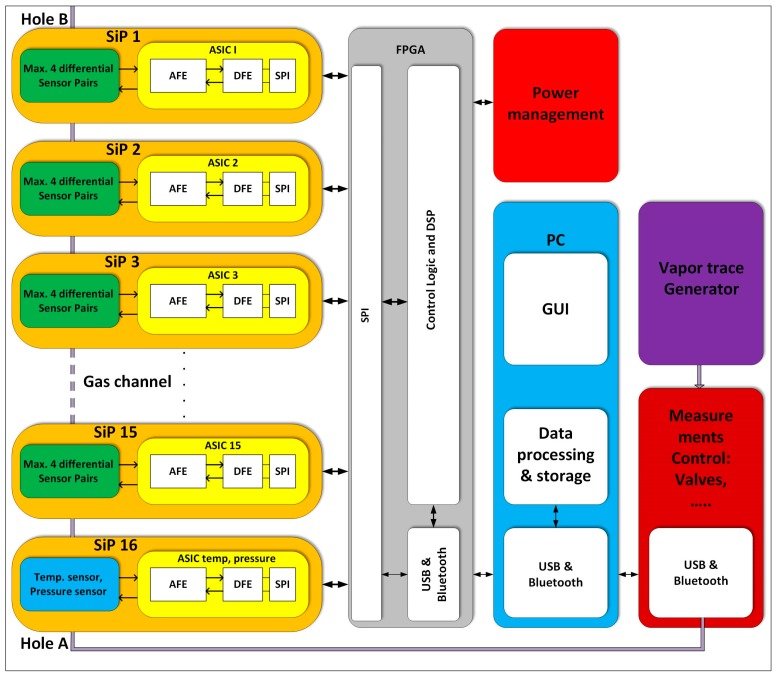
Block diagram which shows 16 SiPs (on the left), each composed of two differently modified sensors (up to 4 possible) and one low noise ASIC. The last SiP contains temperature and pressure sensor and associated signal processing electronics. The DSP is currently implemented on the FPGA together with control logic, and USB/Bluetooth interface. The power management unit deliver appropriate supply voltages for the blocks, while the PC controls the measurement system and gas generator, as well as takes care of additional signal processing, averaging and storage.

A simplified block diagram of a complete detection system is presented in Figure 5. It is composed of 16 Systems in Package (SiPs), where each SiP contains two functionalized sensors: one is a reference sensor, where both functionalized layers are removed, and the second is the active sensor, where only one functionalised surface is removed. In addition, there is a low noise Application Specific Integrated Circuit (ASIC) to detect the capacitance changes. The last SiP (SiP16) contains sensors and corresponding electronics for temperature and pressure measurements in the gas channel.

**Figure 6:**
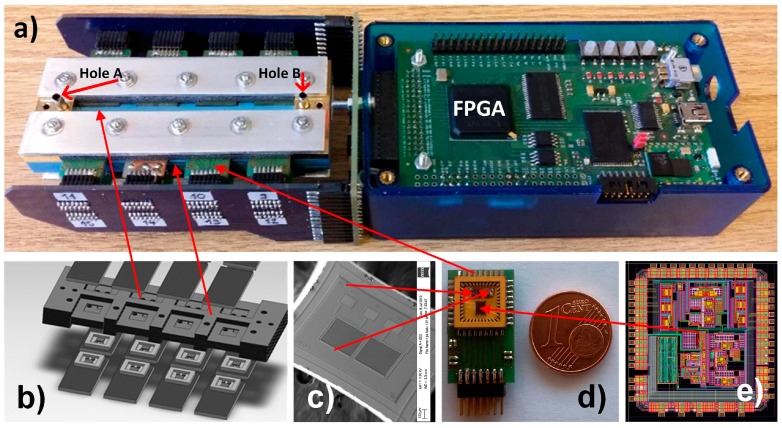
Sixteen-channel vapour trace detection system: (**a**) Implemented detection system without gas generator, where inlets A and B are connected to the gas generator. (**b**) Ceramic gas distribution system and SiP holder, (**c**) One differential sensor, (**d**) SiP composed of one ASIC and two differential sensors, (**e**) layout of the ASIC that implements one channel lock-in amplifier that is capable to process the signals from 4 differential sensors.

We use a ceramic vapour distribution system shown in Figure 6b for distribution of the gas to all sensors in the array and at the same time to hold the SiPs. Exploded views of the detection system are presented in Figure 6b–e. A SEM micrograph of one differential COMB capacitive sensor and a photomicrograph of the ASIC are shown in Figure 6c,e, respectively. The PCB with one ASIC and two differential sensors as a System In Package (SiP) is presented on Figure 6d; one Euro-cent coin is added for comparison. The sizes of the ASIC and the sensors are 2.5 × 2 mm and 1.2 × 1.5 mm respectively. The mixture of gases is delivered via the inlet and outlet holes (Hole A and Hole B) shown in the left side of Figure 6a.

**Figure 7:**
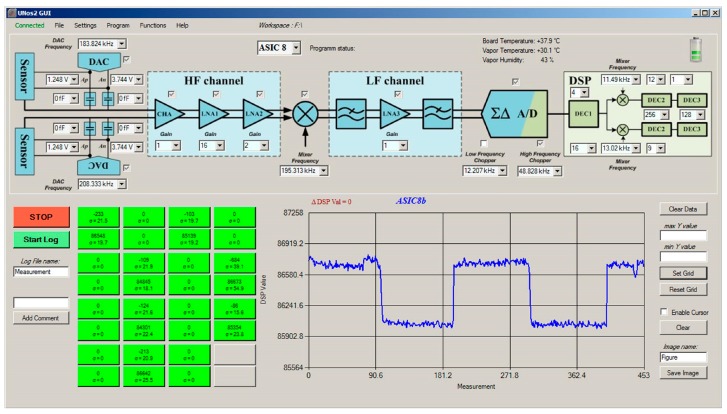
Control screen on the PC, through which it is possible to set the important parameters of the sensors interface, AFE (ASIC) and DSP. A signal from one sensor is presented as a function of time in the lower-right part of the command screen. The responses of all active sensors are presented in real time on the green pixels of the sensor array in the lower left part of the screen. Pixels are differently coloured depending on the real-time value of the signal.

Part of the measurement system is also the PC (computer) with software that controls the detection system and the vapour generator. It adjusts the characteristics of the lock-in amplifier and sensor interfaces implemented in the ASIC, performs specific additional digital signal processing like averaging, presents the results of each channel on the screen, as shown in Figure 7 and stores the results for further processing.

**Figure 8:**
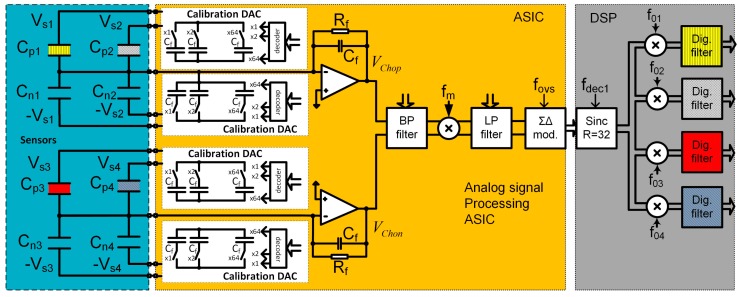
Block diagram of one channel of the detection system. It is composed of the blue region with maximum of 4 differential sensors (different colour represent different modification), the ASIC in yellow region which contains analogue signal processing electronics together with calibration DACs (in white regions), and on the right, in the grey area, the DSP part of the signal processing electronics of one channel.

Before the start of each measurement, each sensor is electronically calibrated in the carrier gas environment. The reason for calibration is two-fold: to calibrate the sensors in the carrier gas, and to reduce the initial capacitance difference of two capacitors in one differential pair. By reducing the difference in capacitance, electronic gain could be maximized and therefor sensitivity of the sensor system in also maximized. A programmable capacitor array is implemented inside the ASIC (see Figure 8) for each capacitive pair and connected in parallel to each differential sensor. Each calibration capacitive DA converter consists of 64 capacitors of approximately 1 fF; the calibration procedure requires one measurement for each differential sensor, before the start of the vapour trace detection using carrier gas. The calibration results are stored in the PC and used before each measurement, adjusting the capacitance difference to be as close to zero as possible. In this way, the dynamic range of each measurement channel is optimized, so larger excitation voltages are possible, which maximizes the sensitivity. 

Each channel implemented in one SiP is composed of a maximum of four differential capacitive sensors. We used only two sensors in the experiments, where the first sensor is a functional sensor, while the second is the reference sensor. The ASIC implements analogue part of four-channel lock-in amplifier and is composed of an Analogue Front End (AFE) and Digital Front End (DFE), control logic and serial interface (see Figure 8). The DSP part performs part of the digital signal processing of the signals. The last element of the whole detection system is the PC, where data from the FPGA arrive via USB or Bluetooth interface. The software on the PC performs additional noise shaping, storage, and presentation. In the future, the pattern recognition algorithms using machine learning and classification [28] will also run on the PC. The detailed description of the function and implementation of one measurement channel are presented in [8,9].

**Figure 9:**
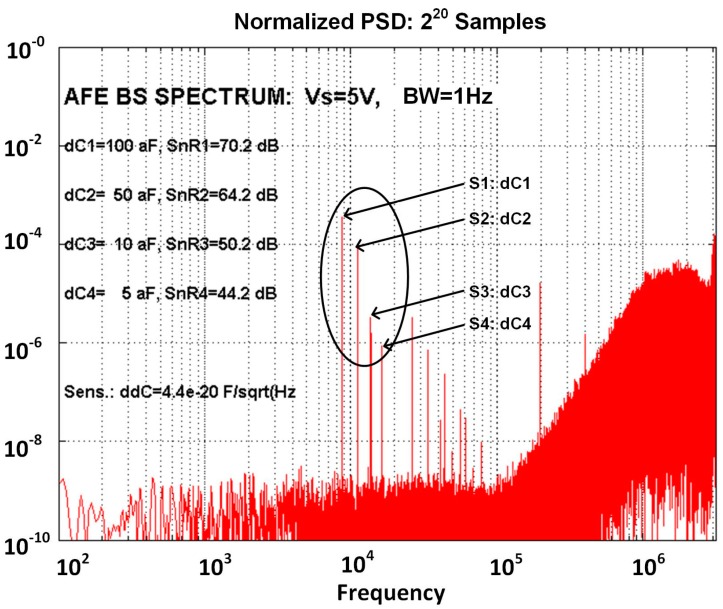
Simulated spectrum at the output of the ASIC with four differential sensors connected to one measurement channel, where each sensor is connected to excitation signal with 5 V amplitude and different frequencies. The amplitudes of spectral components correspond to different capacitance differences; for example the 4th sensor has capacitance difference of 5 aF which corresponds to the smallest spectral line (S4: *dC*_4_ at frequency 16 kHz) with *SnR* of 44 dB, which leads to possible detection sensitivity of 44 zF/$\sqrt{\mathrm{Hz}}$.

Simulated spectrum at the output of the AFE, is presented in Figure 9. It is composed of four spectral lines corresponding to four differential capacitive sensors, where each sensor is excited with different excitation signal frequency; the amplitude of each spectral line is proportional to differential sensor capacitance difference $dC_{i}$, the excitation signal amplitude *V_si_* and the gain in the channel. The smallest spectral component at frequency 16 kHz corresponds to capacitance difference of the 4th sensor with *dC*_4_ = 5 aF, while spectral component at 8 kHz corresponds to the first sensor with *dC*_1_ = 100 aF. Two additional spectral components represent the capacitance difference of the other two sensors (*dC*_2_ = 50 aF and *dC*_3_ = 10 aF). From this spectrum, it is possible to calculate minimum possible detection level, which is based on the signal to noise ratio. For sensor 4 (*dC*_4_ = 5 aF), the associated *SnR*_4_ = 44.2 dB and the detection sensitivity is thus: *ddC*_4_ = 0.044 aF/$\sqrt{\mathrm{Hz}}$; this means, that it is possible to reliably detect the change of the sensor’s capacitance which is bigger than 3*ddC* = 0.13 aF in one Hz band. 

#### 2.3.2. Sensitivity Considerations

**Figure 10:**
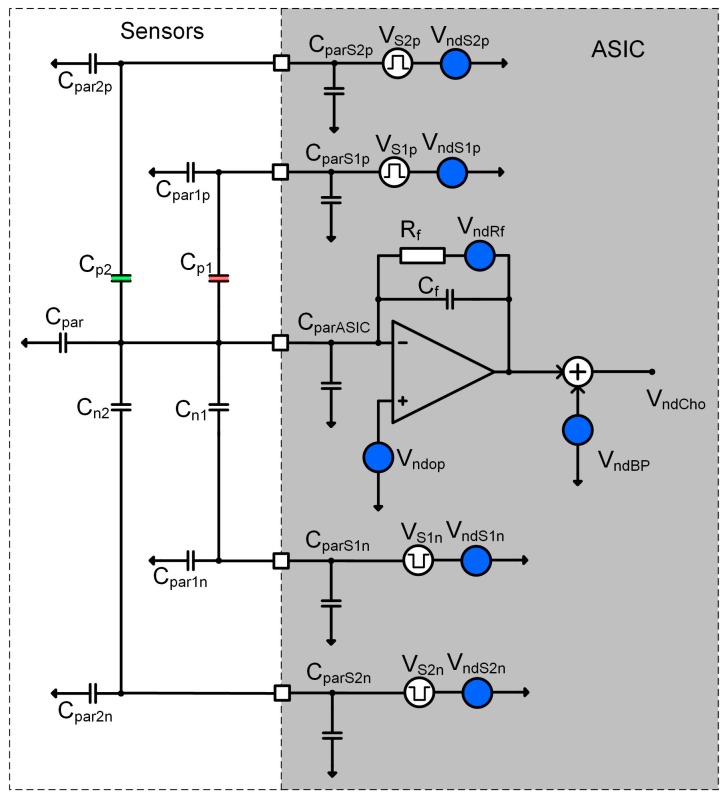
Simplified circuit diagram of one-half of the charge amplifier that includes two differential sensors. Most important parasitic capacitors are marked on that picture together with most important noise sources.

The sensitivity of the entire measurement system is mostly dependent on the sensors and charge amplifier characteristics. Figure 10 shows simplified circuit diagram of one half of the complete charge amplifier. It is composed of an operational amplifier with feedback impedance (*R_f_*, *C_f_*) (to maintain *DC* operating point of the amplifier), and two differential sensors driven by a square wave excitation signals (*Vs1p*, *Vs1n*, *Vs2p*, *Vs2n*) with adjustable amplitudes and frequencies that are higher than 1/f noise corner frequency of the CMOS process used. The main parameter that determines the detection limit is the S/N ratio at the output of the charge amplifier. The elements, which contribute to the noise directly or indirectly are:

- Differential COMB sensor capacitances: (*C_pi_*, *C_ni_*), parasitic capacitances (*C_parip_*, *C_parin_*), the thickness and dielectric constant of the adsorption layer, the distance between the plates, etc.
- The noise level of corresponding charge amplifier around excitation signal frequency (*V_ndop_*, *V_ndRf_*),
- The compatibility of the sensor and the charge amplifier, including parasitic capacitances (*C_parASIC_*),
- The excitation signal generator frequency (*f_si_*), amplitude (*V_si_*) and the noise level (*V_ndSpi_*),
- Input referred noise of all analogue blocks in the chain following charge amplifier (*V_ndBP_*).

Each stage can process the signals from four differential sensors; this can be easily extended to more sensors in the future, being mindful of the noise contributions due to parasitic capacitances. 

Charge amplifier output voltage can be calculated according to (1), where *C_f_* = 0.5 pF is the feedback capacitance of a charge amplifier, ∆*C* is differential sensor capacitance difference observed during the adsorption of the target molecules to the surface of the sensor, and compared to the capacitance when only the carrier gas is present. The *V_s_* is the excitation signal amplitude, while *ω_p_* is the pole frequency caused by *R_f_* and *C_f_* of the charge amplifier block. *H_cha_*(*ω*) = *s*/(*s + ω_p_*) is the signal transfer function of the charge amplifier circuit at corresponding frequency:

(1) $$V_{cho}=V_{s1}\left(\omega {\;}_{1}\right)\cdot \left(\frac{\Delta C_{1}}{C_{f}}\right)\cdot H_{cha}\left(\omega {\;}_{1}\right)+V_{s2}\left(\omega {\;}_{2}\right)\cdot \left(\frac{\Delta C_{2}}{C_{f}}\right)\cdot H_{cha}\left(\omega {\;}_{2}\right)$$

The parameters are selected in such a way, that pole frequency is in a range of 10 Hz and thus much smaller than any of the excitation signal frequencies, which are selected well above the 1/*f* noise corner frequency of the charge amplifier, therefore, 1/*f* noise is neglected in our considerations. The remaining thermal noise power around the excitation signal frequency (*f_s_*_1_ = 195 kHz and *f_s_*_2_ = 204 kHz is responsible for detection noise level and thus for the channel sensitivity. Input referred noise density around 200 kHz reaches *V_nd_op_* < 7 nV/$\sqrt{\mathrm{Hz}}$.

The sensitivity of the detection system is directly dependent on the signal to noise ratio at the charge amplifier output. It can be calculated according to (2) [9], where *V_Sx_* is the excitation signal amplitude, *δCx* is the change of the sensors capacitance because of the adsorption, *C_f_* is the feedback capacitance of the charge amplifier and *∑C_VG_* is the sum of all parasitic capacitances connected to the virtual ground of the charge amplifier:

(2) $$\left(\frac{S}{N}\right)=\frac{V_{Cho}}{\sqrt{2}\cdot V_{ndCho}}≅\frac{V_{Sx}\delta C_{x}}{\sqrt{6}V_{ndop}\left(C_{f}+\sum C_{VG}\right)}$$

The signal to noise ratio thus determine the minimum sensor capacitance change that can be detected and can be calculated according to (3) [9]:

(3) $$\delta C≅3\sqrt{2}\cdot \left(V_{ndop}/V_{Sx}\right)\cdot \left(C_{f}+\sum C_{VG}\right)≅4.5\cdot 10^{-20}\;\left[F/\sqrt{Hz}\right]$$

The system level simulations confirm this sensitivity level, where most important non-ideal effects are included (see Figure 9). The detection sensitivity can be increased using narrower gaps between the combs of the sensor capacitors, increasing the excitation signal amplitude, reducing the thermal noise of the charge amplifier, reducing the feedback capacitance *C_f_* and all parasitic capacitances connected to the virtual ground of the charge amplifier *∑C_VG_*. In this way it is possible to reach the sensitivity down to *dC* = 0.04 aF/$\sqrt{\mathrm{Hz}}$, which is an order of magnitude better than what it is predicted in [9] and currently used in the demonstrator of the detection system.

## 3. Measurements of the E-Nose Responses to Vapour Traces of Explosives and Solvents

The measurements of vapour traces of explosives and solvents in the presence of background molecules such as H_2_O are the most demanding from the sensitivity and selectivity point of view, because the number of targeted molecules is very small compared to the number of all molecules present in the atmosphere. We designed two different measuring setups and several different measuring protocols to test the response of a 16-channels e-nose to different mixtures of explosive vapours and solvents in a background atmosphere. The first setup is based on our vapour generator, which is able to produce well defined concentrations of molecules of different explosives in the carrier gas N_2_. The second setup mimics measuring conditions in more realistic environment and uses spontaneous evapouration of different solvents and toxic gases inside a fume cupboard installed in a closed laboratory room. In the following we shall describe both setups and measurement protocols for both cases.

### 3.1. Experimental Set-Up and Protocols for Measurements Using Vapour Generator

**Figure 11:**
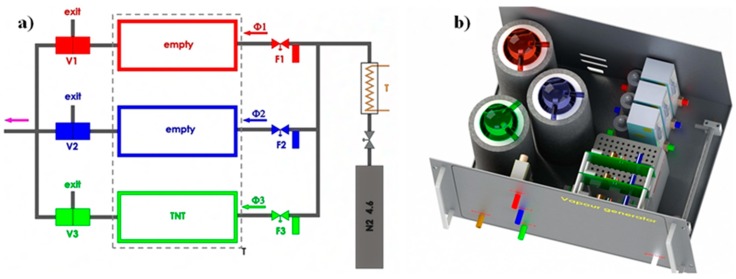
(**a**) Schematic diagram of the vapour generator used for TNT, RDX, DNT and H_2_O measurements. N_2_ gasfrom a storage tank is thermally stabilized (T) and divided into three parallel flow lines (Φ1, Φ2, Φ3), each with electronic flow regulator (F1, F2, F3). The switching between the pure carrier gas and gas with the known concentration of the explosive’s vapour, while keeping the total mass flow constant, is done by mixing the gas from an empty glass cylinder (Φ1) with either pure gas (Φ2) or with saturated gas (Φ3). Three valves V1, V2 and V3 control the mixing. (**b**) A model of the actual vapour generator used in the experiments. Red, blue, and green colours represent individual flow lines.

The vapour generator for different explosives used in these experiments has been described in detail in [8,9]; here we give only some basic information for the sake of completeness. We have developed and tested a computer-controlled vapour generator, which allows long-term and reliable generation of TNT and other vapours of explosives, with concentrations in the range from 5% to 50% of the vapour pressure of selected explosive. The schematic diagram of the device is presented in Figure 11a and a model of a constructed vapour generator in Figure 11b. In our vapour generator a N_2_ carrier gas flow from a storage tank passes through a regulated resistive heater, which heats the flowing gas to a pre-set temperature with an accuracy of ±50 mK. The flow of this thermally stabilized gas is divided into three parallel lines; an electronic flow controller (Aalborg GFC17A, Orangeburg, NY, USA) regulates the gas flow through each line. Each flow line is connected to one of the three equivalent glass vessels, which are thermally stabilized to any pre-set temperature in the range from 25 to 65 °C with an accuracy of ±50 mK. One of the cylinders contains a known mass of the explosive under consideration, which is deposited on a clean piece of glass wool, while the other two cylinders were empty. The generator is programmed to deliver a precisely controlled concentration level of the explosive’s vapour in the output. The vapours of the explosives or other substances of interested are delivered to the e-nose for a pre-determined time period, typically of the order of 10 min. This is then followed by switching the output of the generator to pure carrier gas, which “cleans” the sensors of the e-nose and prepares it for the next sequence. Typical flow rates are of the order of 10 mL/min. 

Great care was taken to avoid any contamination of the system. All components were made out of stainless steel, Teflon, or glass and thoroughly cleaned and flushed with carrier gas for a longer time prior to any usage in the detection experiments. The temperature stability of the output gas with a known concentration of the explosive’s vapour was found to be better than ±1 mK, and the concentration was stable over weeks of constant operation. Several methods for calibration of the concentration of explosive vapours in the output gas were tested. They are thoroughly explained in [9]. 

In addition to the measurements of e-nose response to vapours of various explosives, we also used the vapour generator as a flow-control unit to measure the e-nose response to methane. In this case, a 1.74% mixture of methane in synthetic air was delivered to the e-nose in one cycle and in the next cycle pure synthetic air was delivered to the e-nose. In both cases the flow rates were 10 mL/min.

### 3.2. Experimental Set-Up and Protocols for Sensing Solvents in Ambient Air

**Figure 12:**
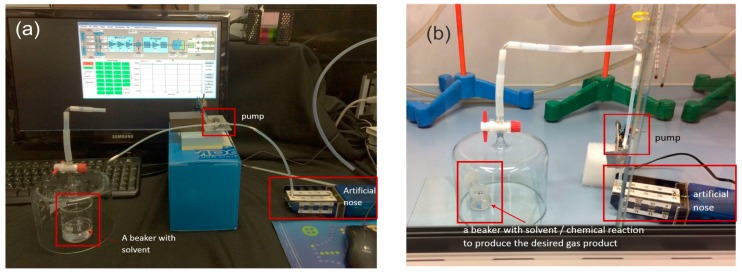
(**a**) The measurement set-up for measuring the response to different solvents in ambient air of the laboratory. (**b**) The set-up for measuring e-nose response to toxic gases in a fume cupboard.

Another set of experiments was performed by sensing solvents (ethanol, methanol, acetone, propane-2-ol, H_2_O, toluene) in the ambient air of the closed laboratory and toxic gases (NH_3_, FeS, HCN, H_2_S) inside a fume cupboard with an exhaust flow rate of 700 m^3^/h. Figure 12(a) shows the set-up for measuring the e-nose response to different solvents in the ambient air of the laboratory. The solvent in the jar is covered, and the inlet the tube of the e-nose pumps the solvent vapours from the tank to a hand operated valve, which delivers the gas either from the air surrounding the experiment or from the glass tank, where the vapours of the solvents are present. We assume to have approximately vapour pressure of the solvent in the tank. The flow rate of the pump is 18 mL/min, the volume of the inlet tube is 8 cm^3^, so it takes approximately some tens of seconds to pump the solvent vapours from the tank to the sensors of the e-nose. One should note that in this experiment the valve selecting the input vapours is hand-operated so the timing of the experiment (i.e., when the solvent vapours are “on” or “off”) is also manual, i.e., there is no clock from the computer.

The response of the e-nose to toxic vapours was measured in a fume cupboard with the exhaust flow rate of 700 m^3^/h. Toxic vapours were produced either by a specific chemical reaction taking place in a beaker, or by placing the solvent in it, as shown in Figure 12b. When measuring responses in the fume cupboard there were rather large changes in temperature and humidity when the cupboard was opened to switch from one phase to another (for example from pure air to air mixed with gas obtained using a chemical reaction). This resulted in significant changes of the background signal because the pressure, temperature and humidity changed when the cupboard was opened also the concentration of the measured gas in the glass dome varied with time, which means that measurements made this way can only be considered as a proof of concept. No absolute quantitative results could be obtained that way. The only information we gained is whether or not the sensors respond to a certain chemical and what their relative responses are like. As the flow rate in the fume cupboard had to be rather large for safety reasons and the system is not completely isolated from the surroundings there may also be a significant difference between the sensors closer to the input of gas into the system.

### 3.3. Signal Extraction and Background Correction

**Figure 13:**
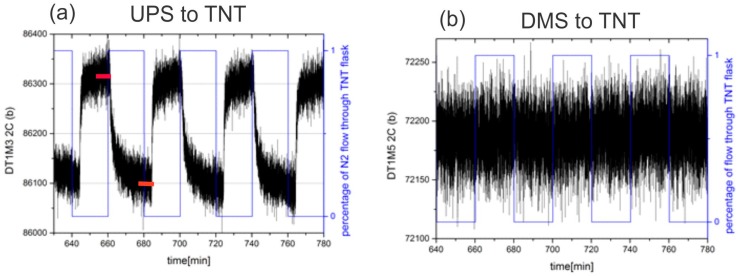
(**a**) Response of **UPS** sensor to switching between pure nitrogen and nitrogen with the maximal concentration of TNT vapour. The red lines indicate measurements that were averaged to determine the amplitude of response. (**b**) Response of **DMS** sensor to switching between pure nitrogen and nitrogen with the maximal concentration of TNT vapour.

Data obtained using the vapour generator was extracted automatically, because the clock of the generator precisely synchronizes the experiment. Typically, the vapours of explosives or methane from the container were delivered through the e-nose for a period of 10 min and then pure N_2_ or synthetic air was delivered for another 10 min period. Two examples of the signal recorded by the **UPS** and **DMS** sensors of the e-nose to TNT vapours are shown in Figure 13a,b.

At the moment, a quite simple algorithm is used to extract the results. From the stored time response, the last 20% of the data in each phase are averaged (see red bars in Figure 13a). The averaged result of each sensor in each phase is stored into appropriate register *R*(*i, Φ_j_*), where *i* is the index of the sensor functionalization and *j* is the index of the phase (*j* = 1,2). The absolute value of the difference of both responses are calculated and stored: ∆*R*(*i*) = |*R*(*i, Φ*_1_) − *R(i*, *Φ*_2_)|.

Measurement results under different conditions are combined by normalizing to the response of the sensor, which shows the maximal response. The normalized response for each sensor in the array is calculated as: *r*(*i,k*) = ∆*R*(*i*)/*max*(∆*R*(*i*), (1,2,..18), where *i* is the index of the sensor (modification layer) and *k* = *1*, *2*, … 14 is the index of the experiment where we used different vapours (TNT, RDX, H_2_O, toluene, etc.). We can compare the responses to different vapours by plotting the relative response of each sensor for each vapour *r*(*i,k*) on several 2D and 3D plots, as presented in Section 3.5. 

### 3.4. Measurement Results

**Figure 14:**
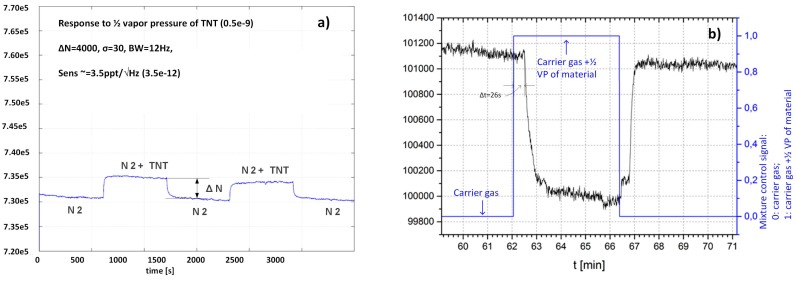
(**a**) Time response of the sensor functionalized with **APhs** to the TNT vapour at ½ vapour pressure of the TNT. (**b**) Time response of the sensor functionalized with **UPS** to H_2_O vapour at approx. vapour pressure. In both cases the response is without dimensions and corresponds to $dC/C_{f}$

Time-dependent responses of two different sensors to different gas composition are presented in Figure 14a for APhS sensor to TNT and Figure 14b for UPS sensor to H_2_O respectively. The results are proportional to relative changes of sensors capacitance difference, when target molecules are adsorbed to the surface of the sensor. 

.

Figure 14a shows time dependent response of the sensor modified with APhS to the gas, which changes from pure carrier gas (N_2_) to the mixture of carrier gas with TNT vapour at 50% of the vapour pressure. From difference of the two readings (∆*N* = 4000), the information about the relative number of molecules of TNT at 50% vapour pressure (0.5 × 10^−9^), the temperature (300 ^o^K), and standard deviation of the noise (*σ* = 30), the sensitivity can be estimated as: *Sens* = 3.5 × 10^−12^/$\sqrt{\mathrm{Hz}}$ (3.5 ppt). The measurement system is fast and can provide as much as 12 readings in one second. However, the measurement itself takes longer time, because of the volume, which must be filled in with. This volume includes all tubes and empy cavities in the ceramic distribution system, which must be filled in before the target molecules arrive to the sensor surface. 

Figure 14b shows the response of UPS modified sensor to H_2_O vapour at room temperature. In this case, the switching of the “gases” was performed automatically; the control signal is represented in blue, while the response of UPS modified senor is presented in the black. During phase Φ_1_ the sensor is exposed to carrier gas, during phase Φ_2_ the sensor is exposed to the mixtures between carrier gas and gas with target molecules. The delay time between the change of the control signal and the response of the sensor is relatively long (∆*t* = 26 s), and is needed to fill-in the empty volume of the tubes, cavities etc. The void volume inside the SiP is negligible (*V_SiP_* ≅ 20 mm^3^) compared to the other volumes.

### 3.5. Responses

**Figure 15:**
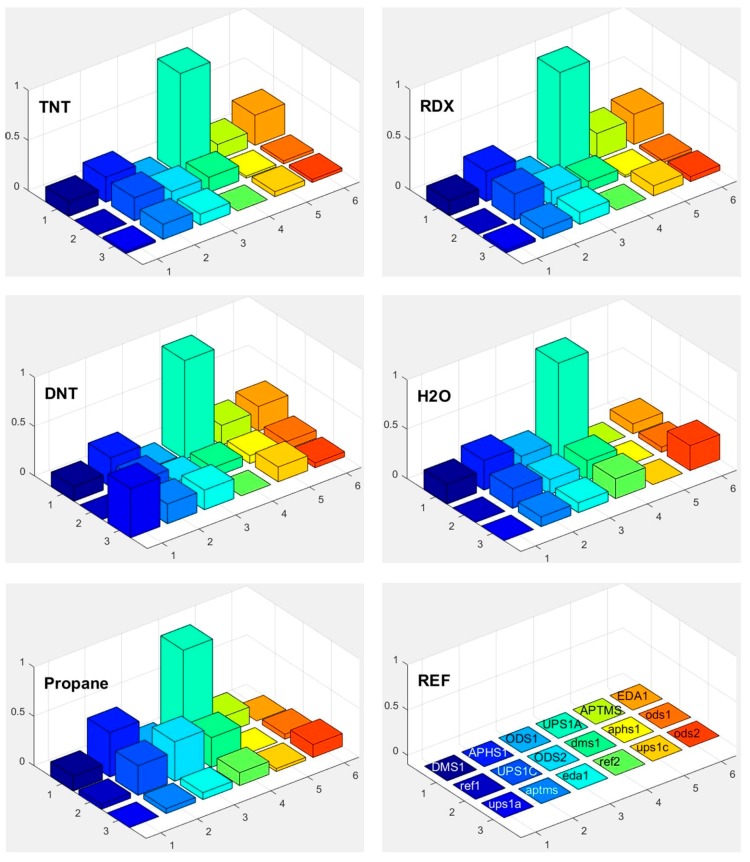
Responses of differently functionalized sensors to vapours of different molecules: TNT, RDX, DNT, H_2_O and propane. The responses were normalized to the response of sensor UPS1A. The positions in the array and different colours of individual pixels shows differently functionalised sensors. The type of the functionalisation layer is printed in the array at the lower right corner (upper case names, for example DMS1, correspond to the active sensor while the lower case names, such as ups1a, correspond to reference sensors, where functionalized layer is removed on both parts of differential sensor).

**Figure 16:**
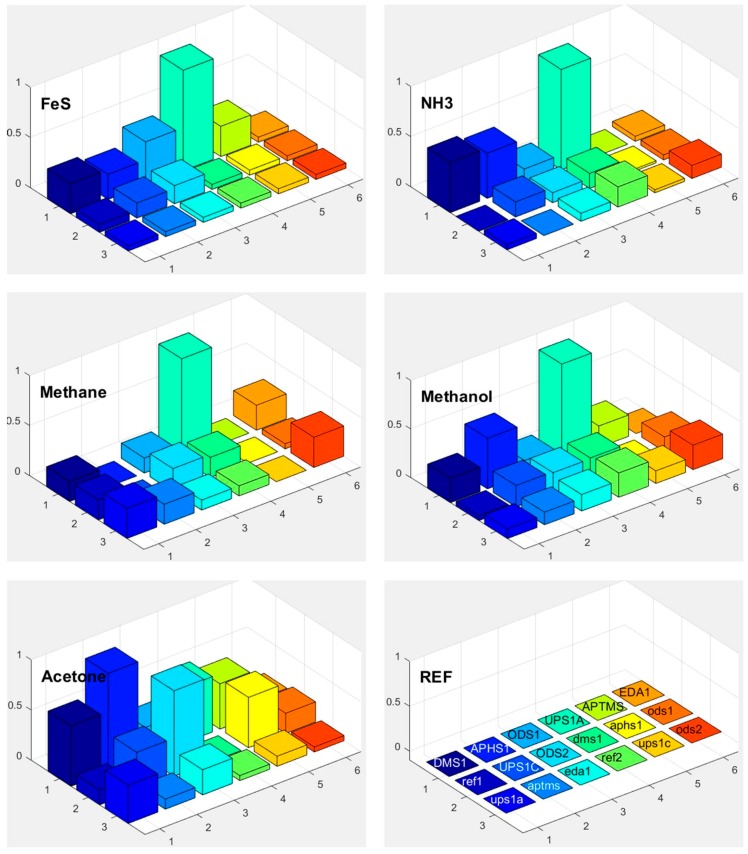
Responses of differently functionalized sensors to vapours of different molecules: FeS, NH_3_, methane, methanol, and acetone. The electronic response of a given sensor was normalized to the response of the sensor UPS1A. The position in the array and the colours of individual pixels shows differently functionalised sensors. The type of the functionalisation layer is printed in the array at the lower right corner (upper case names, for example DMS1, correspond to the active sensor while the lower-case names correspond to reference sensors, where functionalized layer is removed on both parts of differential sensor).

**Figure 17:**
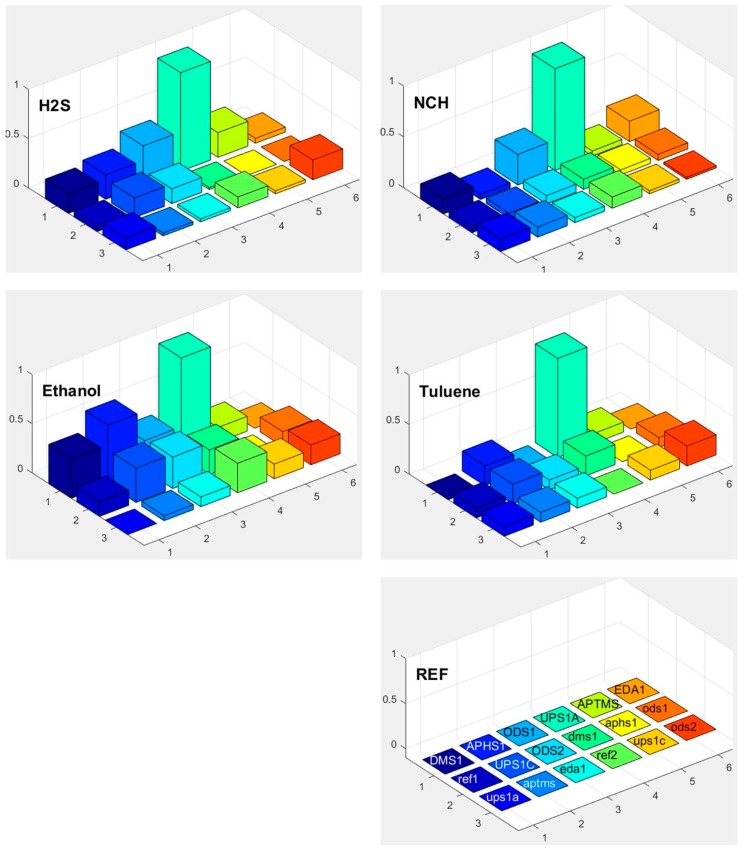
Responses of differently functionalized sensors to vapours of different molecules: H_2_S, NCH, ethanol, and toluene. The electronic response of a given sensor was normalized to the response of the sensor UPS1A. The position in the array and the colours of individual pixels shows differently functionalised sensors. The type of the functionalisation layer is printed in the array at the lower right corner (upper case names, for example DMS1, correspond to the active sensor while the lower case names correspond to reference sensors, where functionalized layer is removed on both parts of differential sensor).

Response of each sensor in the array is presented as a bar in certain location of the array for example (*x* = 1, *y* = 2, for APHS1). Different colours correspond to different sensors with different modification layers; they are marked on the bottom right subplot of Figure 15, Figure 16 and Figure 17. The height of each bar is proportional to normalized relative response (each subplot is marked with the name of the molecule in the gas we use for the measurement). 

**Figure 18:**
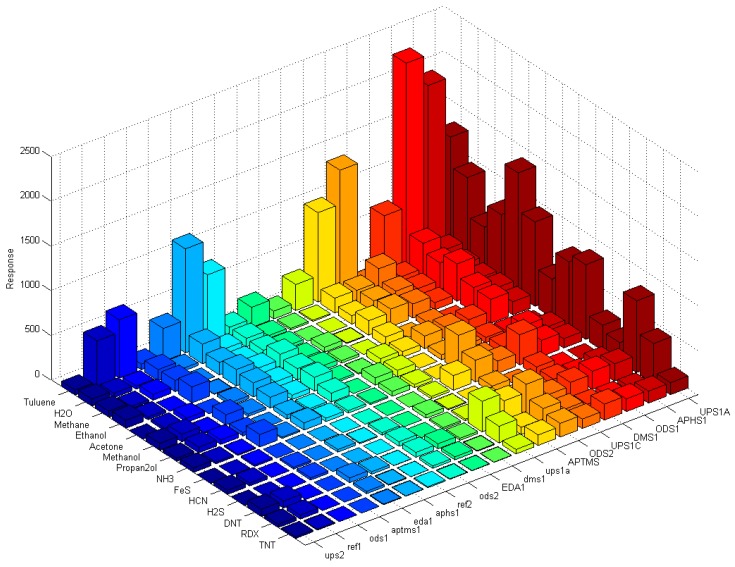
The complete matrix of absolute values of responses of differently functionalized sensors to different vapour traces. Each row on y-axis corresponds to modification layer; the bars are presented in different colour, so each colour presents one modification layer, while each column on x-axis corresponds to different vapour of the experiment. The height of each pixel represents relative response of particular sensor to the particular target molecule in the carrier gas. Sensors with lower case letters (i.e., ups1a) are the reference sensors where the modification layer is removed completely by laser treatment.

Figure 18 shows a matrix of responses of all differently modified sensors to all vapours we used in the experiment. The elements on one axis, say x-axis, correspond to different vapours. The y-axis corresponds to different sensors used in the experiment. The upper case letters (for example UPS1A) define the first active sensor with UPSA modification. The active sensor means that the modification is removed on one part of the sensor, while corresponding reference sensors (where modification layer is removed on the whole sensor) is marked with small case letters (such as ups1a). Such notation is valid for Figure 15, Figure 16, Figure 17 and Figure 18.

### 3.6. Selectivity in Surface Adsorption of Different Vapours

From the relative response plots presented in Figure 18, we can qualitatively say that the 16-channel sensor array of the e-nose provides some selectivity for different vapours. As presented in Figure 15, Figure 16 and Figure 17, the sensors show different relative responses for different vapours. However, it is quite clear that the patterns of the responses are very complex and it is difficult to determine the real selectivity properties of the sensor array. We can get a better insight into the chemical selectivity of different functionalized surfaces by considering the actual number of particular molecules in a given experiment and compare this number to the electric signal measured by the system.

Namely, the number of molecules at a given vapour pressure for different molecules are different for several order of magnitudes. For example, the relative number density of molecules of H_2_O in the carrier gas N_2_ at a water vapour pressure at room temperature is 10^−2^. This means that we have 1 molecule of H_2_O for 100 molecules of N_2_. If we consider the vapour pressure of TNT in N_2_, the relative number density of TNT molecules at a TNT vapour pressure is in a range of 10^−9^. This means that we have one molecule of TNT for 10^9^ molecules of N_2_. The relative number densities of molecules for vapours of H_2_O, TNT and DNT in the carrier gas N_2_, at vapour pressure, together with responses and scaled responses are presented in Table 2.

For example, our APhS modified sensor gives the absolute response of Δ*R* = 1200 (unscaled response), when exposed to the vapour pressure of H_2_O molecules in N_2_ carrier gas. The electronic response of the same APhS sensor to the TNT molecules at vapour pressure is equal to Δ*R* = 4000 (unscaled response). Note that the response to TNT of Δ*R* = 4000 is of the same order of magnitude as the response to water molecules of Δ*R* = 1200. However, the number of H_2_O molecules is much higher than the number of TNT molecules, which means that our APhS sensor is in fact very sensitive to TNT and much less sensitive to H_2_O, because approx. 3.3 × 10^7^ molecules of H_2_O would give approximately the same response as one molecule of TNT. It is clear that the selectivity of our e-nose is actually huge, when detecting TNT compared to the water molecules.

To put the signals on an equal footing by having in mind very different number densities of different molecules, we therefore scale response of each sensor with the number density of molecules in the vapour. We can estimate the preference of adsorption at the surface of particular sensor by comparing scaled electronic responses of the sensors in the array to the vapours of the gas with many molecules. We define scaled response $D_{scaled}$ of a particular sensor as:

(4) $$D_{scaled}=\frac{\Delta R}{\left(N_{s}(X)/N(N_{2})\right)}$$

which is a ratio of the electric response of a given sensor ΔR and the ratio *N_s_*(*X*)*/N*(*N*_2_). Here *N_s_*(*X*) is the number density of target molecules of the type *X* at vapour pressure, while *N*(*N*_2_) is the number density of carrier gas molecules. We introduce a measure *Y*(*x*) for the preference of surface adsorption of molecules of type *X* on a particular sensor:

(5) $$Y=20*\log_{10}\left(\frac{D_{scaled}\left(x\right)}{\min\left(D_{scaled}\right)}\right)$$

**Figure 19:**
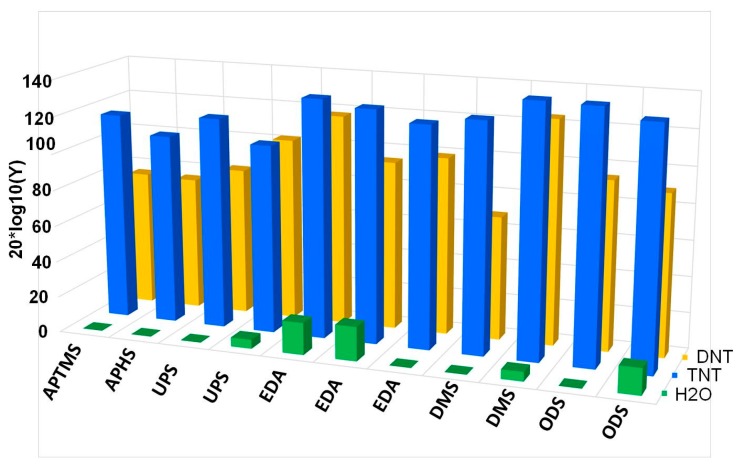
Scaled responses of differently modified sensors to H_2_O, TNT and DNT molecules at vapour pressure. Scaled response to H_2_O molecules is attenuated by factor greater than more than 10^5^ (100 dB) in the worst case.

Here min ($D_{scaled}$) is the smallest scaled response measured in a given set of experiments. Figure 19 shows a 3D bar plot of measures *Y*(*x*) for the preference of surface adsorption of type x molecules (TNT, DNT and H_2_O). We can see that most of sensors used in our experiments have actually a very small response to water molecules, and some sensors attenuate the adsorption of water molecules for more than 120 dB (10^+6^) compared to the scaled response to the TNT. Unfortunately, the water-signal attenuation is still not big enough for reliable detection in the presence of water molecules (and maybe other molecules) on one sensor, because of huge number of these molecules usually present in real ambient conditions. However, the patterns as suggested on Figure 15, Figure 16 and Figure 17 can do the job to improve the selectivity because of different sensitivity of different sensors to different vapours.

Our experiments have also shown that water molecules in the atmosphere background do not block the sensors, and it is possible to detect for example vapour pressure of TNT even in the presence of background water molecules, if the water amount is stable during the experiment. 

### 3.7. Sensor Response at Different Background Water Concentration

In the real environment, there is a huge concentration of water molecules due to natural humidity of the atmosphere and the sensors detect this water quite well. The question therefore is whether this background water influences the response of a given sensor to other molecules, which are added to the carrier gas, containing water vapour. The measurements were performed by using the **EDA** sensor and by slightly changing the flow of N_2_ carrier gas containing TNT vapours through the e-nose system. At the same time, the concentration of water was measured at the **EDA** sensor by a built-in water sensor. The presence of water molecules is difficult to eliminate in the measuring system, because of the diffusion of water from the laboratory atmosphere into the housing, where the sensors are placed. Built-in water sensors allow us to measure the water concentration at the particular sensor and we are therefore able to monitor the presence of water at the sensor. The housing is not vacuum-tight and we noticed that water diffuses from the laboratory atmosphere into the housing and we could clearly detect certain level of background water. Even if the housing were made vacuum tight, thus eliminating diffusion through the housing, there is an outlet pipe, where the vapours are flowing out of the ceramic laminated body of the e-nose. We noticed that there is significant water diffusion through this outlet of the water molecules in the laboratory atmosphere.

**Figure 20:**
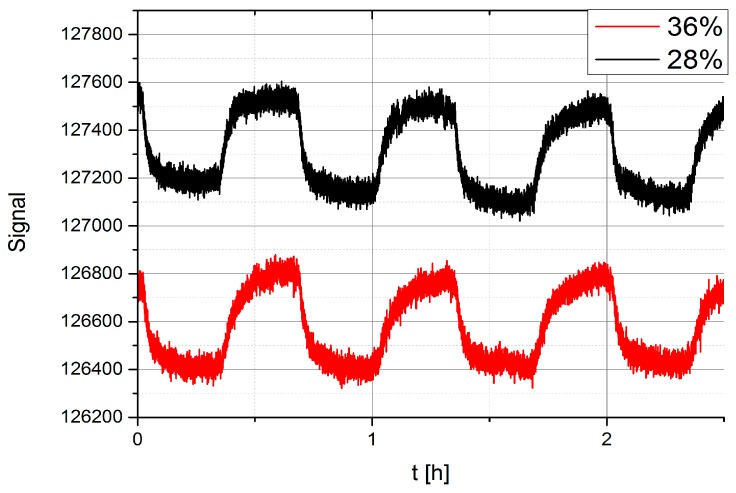
Comparison of the response of the **EDA** sensor to TNT in the presence of different water vapour background concentration of 36% and 28% respectively.

However, we can control the concentration of water molecules in our ceramic housing by changing the flow of the N_2_ carrier gas through the system. When the flow is increased, the N_2_ flushes away the water molecules, replaces H_2_O by N_2_ and therefore changes the humidity around the sensor. Figure 20 shows an example of the **EDA** sensor response to vapours of TNT at two different levels of humidity. The flows in the experiments were 4 mL/min for 28% humidity and 6 mL/min for 36% humidity. The temperature of the TNT flask was held at a constant temperature of 30 °C, which guaranteed a constant TNT vapour concentration that was not affected by the small change of flow.

There is a clear evidence from Figure 20 that the magnitude of the response of **EDA** to vapours of TNT does not change when the humidity is increased from 28% to 36%. One can notice the overall shift of the signal due to different background water, and perhaps a slight change in the shape of the time-response of the **EDA** sensor to TNT, when the water background level is changed.

## 4. Discussion

To our knowledge, our results present one of the first systematic studies of a response of a sensor array to different molecules of explosives, solvents and other biologically harmful simple molecules. This study was performed by using well-defined carrier gas (N_2_, nitrogen) and only a single type of targeted molecules was added to this carrier gas. The results confirm our expectations that even in this most simple case the response of different sensors of our sensor array to this single substance is different, but not to the degree that could allow for clear and unique identification of targeted chemicals. If we consider the complete matrix of normalized responses of differently functionalized sensors to different vapour traces shown in Figure 18, it is clear that there are subtle differences in the response of different sensors to different chemicals. It is also clear that chemical selectivity and the reliability of chemical recognition of a sensor array could be increased by increasing the number of very different sensors. 

While using many differently sensitive sensors is necessary to improve the chemical selectivity of the electronic nose, the problem is how to make a reliable decision on the presence of targeted molecule in the atmosphere? Having N different channels and a multitude of possible combinations of the signals from this N-sensors array, the problem of recognition of a characteristic chemical fingerprint could be solved by using machine-learning approach from the artificial intelligence. Machine-learning methods are very efficient in solving problems where one has to find patterns in large amounts of data and then make decisions. The most famous recent example is Google’s AlphaGo program, which beat the (human) world champion in the game of Go, a feat still considered unsolvable only a couple of years ago. AlphaGo uses deep neural networks to study games already played. Deep neural networks have been experiencing a rapid development in the last couple of years, mostly due to the development of computer architectures that allow parallel processing of data (architecture of graphic cards). Machine-learning methods find use on a broad scope of problems, such as analysis of medical signals and images [29], user habits or forecasting economic trends or identifying the species of bumblebees through analysis of flight buzzing sounds using machine-learning algorithms [28]. In physics, these methods excel in analysis of big data, such as in particle physics and astronomy [30]. 

We propose to use deep-learning methods of the artificial intelligence to teach the N-sensor array of the e-nose to recognize complex and very similar patterns of the matrix of normalized responses and identify hazardous vapours in the atmosphere. Similar to training of dogs to recognize particular chemicals, e-nose could be trained to identify presence of targeted molecules in the atmosphere.

## 5. Conclusions

The conclusions from our results are that there is considerable selectivity and in principle, it is possible to extract the patterns from the matrix of responses, which show with high probability the target molecule response. We propose to apply machine learning methods to the enhance the chemical selectivity of the e-nose. This means that machine-learning algorithms for reliable decision on the presence of targeted molecules should be developed and applied to chemical detection using arrays of large number of different micro-sensors. The goal is real-time processing of signals from multiple channels, encompassing automatic noise reduction, recognizing signals from different measurement setups, combining various channels and analysis using advanced machine-learning methods.

## Figures and Tables

**Table 1:** Surface composition (in atomic %) of SiO_2_ sensors before and after silanization with different silanes (6 h of deposition at 25 °C in acetonitrile).

Sample	C (at. %)	O (at. %)	Si (at. %)	N (at. %)
Reference (blank) sensor	9.9	47.3	42.8	
APTMS	34.9	34.5	25.1	5.5
APhS	33.6	33.4	29.6	3.4
UPS	20.8	42.8	31.8	4.6
EDA	21.6	43.7	32	2.7
DMS	12.1	48.5	38	1.4
ODS	17.5	42.3	40.2	

**Table 2:** Density of molecules for different vapours together with responses and scaled responses of **APhS** sensor.

Vapour	H_2_O	TNT	DNT
Density *N_s_*(*x*)/*N*(*N*_2_)	10^−2^	10^−9^	10^−6^
Response Δ*R*	1.2 × 10^+3^	4 × 10^+3^	1.7 × 10^+3^
Scaled response D_scaled_	1.2 × 10^+5^	4 × 10^+12^	1.7 × 10^+9^
Scaled response relative to H_2_O	1	3.3 × 10^+7^	1.4 × 10^+4^
